# Anæmia in Tropical Diseases

**Published:** 1907-11-16

**Authors:** 


### Tropical Diseases.

### ANÆMIA IN TROPICAL DISEASES.

Many tropical diseases produce anæmia, and though this is generally of the secondary type, nevertheless it is interesting because of the varying features that may be present, and the examination in such cases, apart from the search for specific parasites, is often of great value in diagnosis, and even more so in prognosis.

In malaria, especially when it has lasted for some time, varying degrees of anæmia may be met with. In benign tertian and quartan malaria a reduction of corpuscles follows each paroxysm, but this is often completely made up again in the afebrile intervals between, so that a count, especially if the patient is suffering from a first attack, may show the normal or only a slightly diminished number of red corpuscles per cubic millimetre.

In the pernicious fevers (malignant or æstivo-autumnal malaria) the red corpuscles may go on falling between the paroxysms, and when from faulty treatment or frequent fresh infections a chronic condition supervenes (malarial cachexia) a severe secondary anæmia results. The erythroblasts may sink below 2,000,000 per cm., or 40 per cent. of the normal; there is deformity in their shape (poikilocytosis), an irregularity in their size (microcytes and megalocytes), and in their staining (polychromatophilic and basophilic degeneration). Nucleated forms may be found (normoblasts, *e.g.* red corpuscles of a normal size but with a nucleus), with megaloblasts (red cells of a larger size than normal with a nucleus) occasionally in the worst cases. The hæmoglobin is diminished usually proportionately to the corpuscles; the leucocytes are normal or diminished in number, the polymorphonuclear and large mononuclear ones often carrying the characteristic melanin pigment; differentially there is generally a relative increase in the large mononuclear leucocytes, counts of 10 to 20 per cent. being common. (Normally those form only 5 per cent. or under of the total.) The following counts illustrate those points:—

I. Double benign tertian infection. Relapse.

R., 3,740,000; W., 9,300; Hb., 80 %.

*P.M.N., 56 %; L.M., 17 %; l., 20 %; E., 2 %; T., 4 %; M., 1%.

II. Double benign tertian infection. Slight relapse after good treatment.

R., 5,000,000; W., 8,600; Hb., 96 %.

P.M.N., 58 %; L.M., 4 %; l., 31 %; E., 2 %; T., 4 %; M., 1 %.

III. Malignant infection. Cachexia.

R., 2,430,000; W., 5,000; Hb., 50 %.

P.M.N., 49 %; L.M., 10 %; l., 30 %; E., 5 %; T., 5 %; M., 1 %.

IV. Malignant infection, old relapse after good treatment, a few crescents in blood.

R., 4,530,000; W., 8,600; Hb., 90 %.

P.M.N., 57 %; L.M., 4 %; l., 31 %; E., 7 %; T., 1 %.

Note. Almost complete restitution of reds, large mononuclear increase disappeared.

The changes found in the blood in kala-azar resemble very closely those seen in "splenic anæmia." There is a marked secondary anæmia and a corresponding reduction in the hæmoglobin with a much more marked leucopenia than in malaria; the Leishman bodies may be detected in the leucocytes from the peripheral blood, and the large mononuclear elements are increased. Nucleated red corpuscles are common, and poikilocytosis, etc., is present.

I. R., 2,600,000; W., 1,900; Hb., 55 %.

P.M.N., 45·5 %; L.M., 11 %; l., 39 %; E., 1 %; T., 3·5 %.

Nucleated reds present in proportion of 1 to 300 leucocytes. Note the anæmia, the very great diminution of the whites, and the slight increase in the large mononuclear leucocytes.

II. R., 2,590,000; W., 2,800; Hb., 46 %.

P.M.N., 44 %; L.M., 21 %; l., 33 %; E., 0 %; T., 2 %.

Nucleated reds in proportion of 2 to 300 leucocytes.

In Trypanosomiasis varying grades of anæmia are met with not differing much from those of malaria: very careful search should reveal the specific trypanosome.

R., 2,800,000; W., 5,000; Hb., 45 %.

P.M.N., 41·2 %; L.M., 17·8 %; l., 35·7 %; E., 1·8 %; T., 2·5 %; M., 1 %.

Trypanosomes in proportion of 2 to 300 leucocytes.

In this case there is marked anæmia, the leucocytes are slightly reduced, but not so markedly so as in kala-azar (as a rule they are just under or about normal); the large mononuclears are increased; nucleated reds are not common; poikilocytosis, though present, is not extreme, and degenerative changes (polychromatophilia) are met with.

Any prolonged suppuration will produce a grade of anæmia, and tropical abscess is no exception to the rule. There is nothing specially peculiar about the red corpuscles; poikilocytosis may show itself, but is not marked, and nucleated red corpuscles are seldom met with. The leucocytes are increased absolutely, a very important point in the differential diagnosis of this disease from those already mentioned. The polymorphonuclear leucocytes are the ones that make up this augmentation, and this should always put one on guard. The following is the count of the blood of a man who had a tropical abscess in the upper part of the right lobe of the liver which ruptured into and discharged through the lung.

R., 2,980,000; W., 17,200; Hb., 60 %.

P.M.N., 82 %; L.M., 2 %; l., 10 %; E., 2 %; T., 2 %; M., 2 %.

Note the anæmia and the leucocytosis.

Ankylostomiasis is the most potent factor in the production of anæmia in the tropics, and failure to detect it has often resulted in disastrous consequences. Seen at home by the general practitioner it is usually confused with pernicious anæmia, a serious mistake, because the treatment of the two complaints is so different. Varying grades of anæmia may be met with, depending on the severity of the disease and other factors. An average red count is between two and three million reds. The hæmoglobin by some is said to follow the chlorotic type, but in the counts appended it will be seen to be only proportionally

* P.M.N. = polymorphonuclear. L.M. = large mononuclear. l. = lymphocytes. E. = eosinophile. T. = transitional. M. = mast cells.

diminished. The leucocytes are at first normal, then later may be increased somewhat. The eosinophile leucocytes are relatively increased (18 to 20 per cent. of the total). A mild case of ankylostomiasis:—

R., 4,480,000; W., 11,000; Hb., 83 %.

P.M.N., 42 %; L.M., 3 %; L., 36 %; E., 15 %; T., 3 %; M., 1 %.

Note the slight anæmia, the almost equal reduction of hæmoglobin, the increase of the eosinophiles.

A severe case:

R., 1,600,000; W., not counted; Hb., 30 %.

Note the anæmia. Equal reduction of hæmoglobin.

Varying grades of anæmia, depending on the severity of the case, are again met with in bilharziosis.

Case with severe symptoms, profuse hæmorrhage from bladder:

R., 3,320,000; W., 10,600; Hb., 60 %.

P.M.N., 56 %; L.M., 10 %; L., 20 %; E., 14 %; T., 0.

Note the anæmia, slightly increased leucocytes, equal diminution of Hb, and increase of eosinophiles.

